# Intestinal Form of Human Coronavirus 229E Plays No Role in Peritoneal Sclerosis Pathology in Dialysis Patients

**DOI:** 10.1155/av/2172144

**Published:** 2025-04-01

**Authors:** Sirwan Sleman

**Affiliations:** Department of Microbiology, College of Veterinary Medicine, University of Sulaimani, Sulaymaniyah, Iraq

**Keywords:** coronavirus 229E, encapsulating peritoneal sclerosis (EPS), pan-human coronavirus PCR, peritoneal dialysis (PD), viral peritonitis

## Abstract

Infectious peritonitis is found to be a leading factor in the development of viral peritonitis (VP) and encapsulating peritoneal sclerosis (EPS) in patients undergoing peritoneal dialysis (PD) treatment. Bacterial and fungal infections are a major cause of peritonitis in PD patients. Viral infections have rarely been reported in association with peritonitis in PD patients; about 20% of cases are fungal and bacterial culture-negative (so-called sterile peritonitis). Several possible viral causes are reported to cause peritonitis, Coronaviruses are an important virus group that has been found to cause peritonitis in animals (cats), although in human beings these viruses have not been reported to associate with peritonitis. The purpose of this study was to investigate whether the intestinal form of Human 229E coronavirus plays a role in peritonitis and EPS for several peritoneal fluid samples collected from patients with confirmed EPS. Thirty-seven peritoneal fluid samples from 12 patients with histologically confirmed EPS from Manchester University Hospitals were extracted using QIAamp RNA Mini Kit to purify viral RNA. The purified RNA was reverse transcribed and tested using a pan-coronavirus PCR designed to pick up all known human and animal coronaviruses. None of the peritoneal fluid samples was positive, suggesting that active coronavirus infection is not associated with the development of VP in dialysis patients.

## 1. Introduction

Peritoneal dialysis (PD) is the main type of dialysis that is commonly used today and is usually the first choice of treatment for patients with acute kidney failure disease. It is performed by flushing out the accumulated liquids and dissolved substances from the blood across the patient's peritoneal membrane. This is either performed by automatic PD in which the accumulated fluid is drained out and eliminated via a permanently inserted catheter into the peritoneal cavity overnight or by continuous ambulatory PD which allows constant daily exchanges [1].

PD is usually associated with significant risks and complications. The most obvious complication is peritonitis which usually leads to encapsulating peritoneal sclerosis (EPS) [2].

EPS is a rare and serious complication of PD that is associated with high morbidity and mortality. It is characterized by the presence of extensive fibrosis and sclerosis of the peritoneal membrane with severe adhesion of the visceral organs that results in reduced ultra-filtration with intestinal obstruction, sepsis, and finally death if left untreated. The exact etiology and pathogenesis of this syndrome are not well understood. Among several factors, the infection may play a proportional role in the occurrence of this syndrome [3, 4].

Infectious peritonitis remains an obvious cause of peritoneal inflammation and fibrosis and is one of the leading predisposing factors found to be associated with EPS, especially in severe cases following catheter loss and shifting to hemodialysis.

Bacteria and fungi are generally the most common cause of PD–related infectious peritonitis [5]. Whilst viruses are very rarely found to be associated with peritonitis, the probability of a viral involvement should always be taken into account in patients diagnosed with culture-negative peritonitis, particularly when antibiotic treatment fails to cure the infection [6]. In recent years, a few studies of viral infections have been reported to cause viral peritonitis (VP) in patients on PD such as coxsackievirus B1 [7], cytomegalovirus [8], and herpes simplex viruses [9]. Furthermore, in an animal model, parapoxviruses have been found to produce vascular endothelial growth factor (VEGF) which stimulates angiogenesis [10]. All these indicate that viruses can infect the peritoneal membrane and induce the proliferation of blood vessels and fibrous tissue formation which may then lead to the occurrence of EPS.

In general, the viral cause of peritonitis requires further studies because there might be other viruses to infect the peritoneum during PD and predispose patients to EPS, especially those viruses affecting the gut. The most important example is coronaviruses which can cause infection in both the respiratory and intestinal tract of humans as well as in animals. Particularly in animals, a feline coronavirus has been found to cause peritonitis with ascites and has been associated with a condition called multiple pyogranuloma [11–13]. Although this condition is different from EPS in humans, it gives rise to the possibility that human coronaviruses may play a role in the development of peritonitis.

## 2. Materials and Methods

### 2.1. Chemicals and Reagents

  QIAamp RNA Mini Kit (50) (QIAGEN, West Sussex, United Kingdom)
  RNase-free water (PCR water) (Promega Corporation, Madison, Winconsin, United States of America)
  Power SYBR Green RNA-to-CT 1-Step Kit:
  Power SYBR Green RT-PCR Mix (2x) (SYBR Green I dye, AmpliTaq GoldDNA Polymerase, Ultra Pure (UP), dNTPs, ROX passive reference, and optimized buffer components) (Applied Biosystems, Warrington, United Kingdom).
  Reverse Transcriptase Enzyme Mix (125x) (ArrayScript UP Reverse Transcriptase and RNase inhibitor) (Applied Biosystems, Warrington, United Kingdom).
  Human coronavirus P3 Forward primer 20 μM (Eurofins MWG Operon, Ebersberg, Germany).
  Human coronavirus P3 Reverse primer 20 μM (Eurofins MWG Operon, Ebersberg, Germany).
  Human coronavirus 229E (Eurofins MWG Operon, Ebersberg, Germany).

### 2.2. Equipment

Step OnePlus Real-Time PCR System (Applied Biosystems, Warrington, United Kingdom), MicroAmp Fast Optical 48-Well Reaction Plate (Applied Biosystems, Warrington, United Kingdom), MicroAmp Optical 8-Cap Strip (Applied Biosystems, Warrington, United Kingdom), and pipettors (0.5–1000 μL) (Gilson Inc., Middleton, Wisconsin, United States of America).

### 2.3. Primers

A pair of oligonucleotide primers complementary to different regions of the coronavirus genes was used for real-time PCR amplification. The detail about primers is shown in Table 1.

### 2.4. Sample Collection

Thirty-seven peritoneal fluid samples were collected from 12 patients with histologically proven EPS at the Manchester University Hospitals. Diagnosis of EPS was made around functional (symptoms) and structural (scans) criteria according to the International Society of PD (IPSD) guidelines. The diagnosis of EPS in all cases was based on clinical suspicion confirmed with, primarily, radiologic findings (computerized tomography scans). Pathologic confirmation was obtained at the time of laparotomy for the management or catheter removal. All samples were stored at −80°C in the renal transplant laboratory prior to their use in this laboratory and they were only being thawed when the examination was carried out. Two samples of peritoneal fluid were obtained from patients without symptoms of EPS to serve as controls.

### 2.5. Sample Extraction

From each diluted peritoneal fluid specimen, 200 μL was used for extraction and purification of nucleic acid. QIAamp RNA MiniElute Virus Spin Kit and microcentrifuge are used for RNA extraction according to the protocol for the purification of viral nucleic acid from plasma or serum.

### 2.6. Standard for Coronavirus PCR

#### 2.6.1. Coronavirus Plasmid

The sequence matching the coronavirus primer and probe targets was inserted into a pEX-A plasmid, this was cultured in *Escherichia coli,* and the plasmid was then selected and purified. The details about the plasmid are shown in Table 2.

The plasmid was supplied lyophilized. The plasmid was resuspended in TE buffer to give a concentration of 1 μg/μL and stored at −80°C until use.

#### 2.6.2. Copy Number/mL Calculation

An estimation of the copy number per mL was made based on the molecular weight of the plasmid and the quantity supplied.

### 2.7. Coronavirus PCR

A reverse transcriptase reaction mixture of 15 μL was 2 μL 10X RT buffer, 4.4 μL magnesium chloride, 4 μL dNTPs, 0.4 μL RNase inhibitor, random hexamers 1 μL, 0.5 μL MultiScribe, and 2.7 μL nuclease-free water. A 5 μL of the extract was then added to this mixture and run on the thermal cycler at 48°C for 30 min and 95°C for 10 min.

A PCR reaction mixture of 20 μL was subsequently prepared from 10 μL of SYBR Green master mix, 2.5 μL from each human coronavirus P3 forward and reverse primers (20 μM), and 4 μL of RNase-free water. 5 μL from the pEX-A template, sample, or negative PCR controls were added. The real-time PCR thermal cycling parameters were as follows: 40 cycles of 95°C for 15 s of denaturation followed by 57°C for 1 min for annealing and extension and then for the melting curve: 95°C for 15 s of denaturation followed by 57°C for 15 s of annealing and later 95°C for 15 s of denaturation. Once the PCR run had finished, data from the amplification plot and melting curve were analyzed and examined.

## 3. Results

### 3.1. Real-Time PCR Assay Optimization for Coronavirus

**Figure 1:**
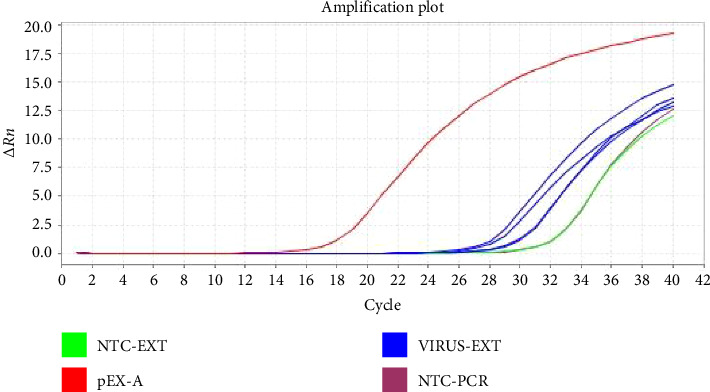
The amplification plot for a PCR run shows Ct values for extracted coronavirus, positive control (pEX-A), and negative extraction (NTC-EXT) with negative PCR controls (NTC-PCR) at a cycling condition of 57°C.

**Figure 2:**
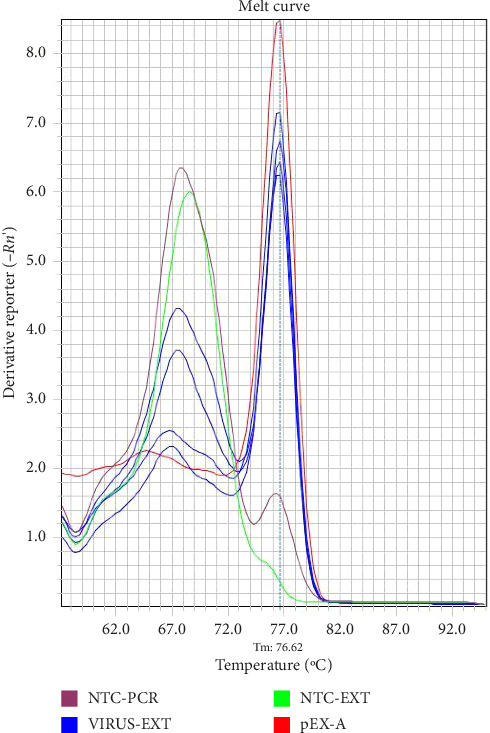
The derivative melting curve for a PCR run shows Tm for extracted coronavirus (VIRUS-EXT), positive control (pEX-A), and negative extraction (NTC-EXT) with negative PCR controls (NTC-PCR) at a cycling condition of 57°C.

Test samples containing 680 copies/μL of the plasmid template or Coronavirus were tested using three different primer concentrations (20, 10, and 5 μM) and three different annealing/extension temperatures of 57°C, 59°C, and 60°C to derive the optimum cycling conditions. Examples of amplification plots and melting curve analysis are shown in Figures 1 and 2.

### 3.2. Sample Inhibition

A 100 μl peritoneal fluid sample was spiked with 100 μl of coronavirus 229E bringing all to about 200 μl. No sample inhibition was found.

### 3.3. Pan-Coronavirus PCR

The pan-coronavirus PCR was designed to detect all known animal and human coronaviruses through the use of two primer sets within a single assay. According to the data from the melting curve and amplification plot generated by real-time PCR assay, we did not detect coronavirus in all the 37 peritoneal fluid samples; this suggested that there is no active coronavirus infection in patients who develop EPS. The details about the samples and results are shown in Table 3.

The specific amplifications were reviewed by amplification plots by checking the threshold cycle (Ct) values produced from each specimen, whilst the melting curve analysis was used to determine the positivity of samples that is it was used to differentiate specific PCR products from nonspecific amplification by looking at the melting point (Tm) of the samples as the nonspecific amplifications have a lower melting temperature (Tm) in comparison to positive (specific) amplifications. The Tm for coronaviruses ranged between 75.5°C and 80.0°C, that is, the Tm for the positive samples should lie within this range.

## 4. Discussion and Conclusion

In animals particularly cats, a strain of coronavirus called feline coronavirus has been reported to infect a wide spectrum of organs including the intestine and peritoneum. The virus can replicate in the gut and cause intestinal infection known as feline infectious enteritis, which in severe cases may lead to intestinal perforation and peritoneal inflammation known as feline infectious peritonitis. The disease is usually complicated and may be associated with ascites and vasculitis which will eventually progress to multiple granulomatous lesions called intestinal pyogranuloma [11–16]. Indeed, this fibrotic lesion is different from EPS histopathologically, but it still raises the question that the coronaviruses in humans may also cause peritonitis and peritoneal fibrosis which are described as the leading factor for EPS since the novel human coronaviruses (SARS-CoV, MERS-CoV, and SARS-CoV-2) have been reported to infect the intestine and cause diarrhea in humans, although respiratory tract infection was regarded as the main manifestation of coronaviruses [17–19]. This is, in addition to the possibility that coronaviruses may associate with the development of EPS where the viruses may trigger EPS as a result of a “hit and run” infection event. Therefore, it was necessary to investigate whether human coronaviruses participate in infectious peritonitis and ESP, which are rare but fatal conditions if left untreated.

In our project, based on the results from real-time PCR assay amplification plot and melting curve analysis, we identified that the novel human coronaviruses in all examined peritoneal fluid samples available from patients with confirmed EPS were not associated with coronavirus infection, that is, the active coronavirus may not play a role in patients who develop peritoneal pathology (EPS). Collectively, our data indicate a piece of evidence that there is no relationship between human coronavirus and peritoneal pathology in patients undergoing dialysis.

### 4.1. Study Limitation

Although our study shows that human 229E coronavirus plays no role in ESP, some limitations of our study should not be excluded. First, this study is performed on limited numbers of random cross-sectional peritoneal fluid specimens collected from confirmed EPS patients from a specific area (from around the United Kingdom) and coronavirus infections in humans are generally not a common infection in the world, although new cases of this virus are still emerging especially with novel coronaviruses. Another point is that because EPS is the end-stage pathology, the virus might be cleared by the immune system raised against the virus at this stage of the syndrome, thereby giving a false-negative result. Therefore, the development of pan-coronavirus serological tests will be necessary to further prove whether coronavirus has any role in the development of EPS or not.

## Figures and Tables

**Table 1:** Detail about primers.

Oligonucleotide name	Sequences 5′–3′	Length	Tm (°C)	Molecular weight	GC (%)	Vol. for 100 pmol/μL
Human coronavirus P3 forward primer	5′TGGGGAGTAATGAACCCGGTAATGT′3	(25)	63	7786	48	251
Human coronavirus P3 reverse primer	5′ACATGTAAAAGAGCTAATAACAC′3	(23)	53.5	7057	30.4	241

**Table 2:** Details about plasmid.

Supplier	Eurofins
Plasmid name	pEX-A-coronavirus standard
Gene name	Coronavirus standard
Vector backbone	pEX-A
Cloning	Vis restriction type IIS restriction enzymes
Designation	5. coli DH5alpha
Internal name	U984-2-A-D
Antibiotic selection	Ampicillin
Gene size	78 bp
Plasmid size	2450
Quantity	42 μg

**Table 3:** Details about samples, patients, and results.

Patient no.	Collected specimen number	Patient reference	Date of specimen	Result
1	17	052	28/03/2008	< 42 copies/mL

2	2	G01 001	07/07/2004	< 42 copies/mL
	7	G01 001	13/12/2002	< 42 copies/mL
	8	G01 001	17/06/2003	< 42 copies/mL
	12	G01 001	16/12/2003	< 42 copies/mL
	14	G01 001	18/09/2002	< 42 copies/mL

3	35	G01 041	18/06/2002	< 42 copies/mL
	11	G01 86	16/12/2003	< 42 copies/mL
	32	G02 122	07/09/2005	< 42 copies/mL
	15	G02 52	02/10/2002	< 42 copies/mL

4	1	G03 016	06/12/2006	< 42 copies/mL
	19	G03 016	09/07/2008	< 42 copies/mL
	20	G03 016	03/07/2006	< 42 copies/mL
	29	G03 016	05/01/2005	< 42 copies/mL
	31	G03 016	29/06/2005	< 42 copies/mL

5	9	G03 18	26/04/2004	< 42 copies/mL
	10	G03 18	22/04/2003	< 42 copies/mL
	27	G03 18	03/12/2003	< 42 copies/mL

6	13	G03 92	12/09/2004	< 42 copies/mL
	18	G05 018	28/05/2004	< 42 copies/mL

7	16	G05 10	08/04/2003	< 42 copies/mL
	28	G05 10	03/02/2004	< 42 copies/mL

8	21	G05 134	07/09/2007	< 42 copies/mL
	22	G05 134	16/08/2005	< 42 copies/mL
	26	G05 134	27/02/2006	< 42 copies/mL

9	24	G05 18	03/12/2003	< 42 copies/mL
	25	G05 18	28/05/2004	< 42 copies/mL
	34	G05 18	23/04/2003	< 42 copies/mL

10	23	G05 29	22/05/2003	< 42 copies/mL

11	3	G05 72	28/08/2003	< 42 copies/mL
	4	G05 72	18/03/2004	< 42 copies/mL
	5	G05 72	06/10/2004	< 42 copies/mL
	6	G05 72	26/08/2008	< 42 copies/mL
	33	G05 72	14/06/2007	< 42 copies/mL

12	30	G122	06/01/2006	< 42 copies/mL

Non-disease controls	36	40	10/03/2004	< 42 copies/mL
	37	G01 123	12/05/2005	< 42 copies/mL

## Data Availability

The datasets used and/or analyzed during the current study are available from the corresponding author on reasonable request.
